# The aetiology of cardiovascular disease: a role formitochondrial DNA?

**DOI:** 10.5830/CVJA-2017-037

**Published:** 2018

**Authors:** Venter Marianne, H van der Westhuizen Francois, L Elson Joanna, L Elson Joanna

**Affiliations:** Human Metabolomics, North-West University, Potchefstroom, South Africa; Human Metabolomics, North-West University, Potchefstroom, South Africa; Human Metabolomics, North-West University, Potchefstroom, South Africa; Institute of Genetic Medicine, Newcastle University, UnitedKingdom

**Keywords:** mitochondrial DNA, cardiovascular disease, MutPred, mutational load, African

## Abstract

Cardiovascular disease (CVD) is a world-wide cause of mortality in humans and its incidence is on the rise in Africa. In this review, we discuss the putative role of mitochondrial dysfunction in the aetiology of CVD and consequently identify mitochondrial DNA (mtDNA) variation as a viable genetic risk factor to be considered. We then describe the contribution and pitfalls of several current approaches used when investigating mtDNA in relation to complex disease. We also propose an alternative approach, the adjusted mutational load hypothesis, which would have greater statistical power with cohorts of moderate size, and is less likely to be affected by population stratification. We therefore address some of the shortcomings of the current haplogroup association approach. Finally, we discuss the unique challenges faced by studies done on African populations, and recommend the most viable methods to use when investigating mtDNA variation in CVD and other common complex disease.

## Mitochondrial DNA

Cardiovascular disease (CVD) remains the main noncommunicable cause of morbidity and mortality in humans.^1^ While environmental factors and lifestyle choices play a major role in CVD, it is also recognised that genetic factors contribute significantly to the aetiology thereof. In this regard, several studies, most recently genome-wide association studies (GWAS), have contributed to identifying genetic loci involved in CVDs, and their association with behavioural and biological risk factors.^2^-^7^

Despite the numerous nuclear DNA (nDNA) variants identified, only a small portion of the heredity of CVDs can thus far be accounted for by variants discovered with GWAS studies.^8^ For instance, the 46 loci identified for coronary artery disease (CAD) only account for about six to 13% of CAD hereditability.^9^-^11^

**Fig. 1:**
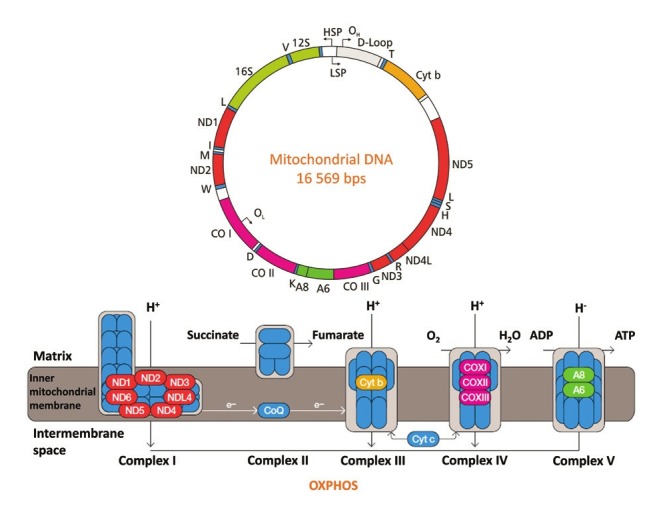
mtDNA encodes for 22 tRNA and two rRNA molecules, as well as 13 polypeptide sub-units of the OXPHOS enzyme complexes, as indicated by colour. Enzyme complexes I–IV are involved in a series of redox reactions, which transfer electrons from carriers nicotinamide adenine dinucleotide (NADH) and flavin adenine dinucleotide (FADH2) to oxygen molecules. During these catalytically favourable reactions, H+ ions are pumped from the mitochondrial matrix into the mitochondrial intermembrane space to create a proton-motor force across the inner mitochondrial membrane. This force is used by complex V to catalyse the phosphorylation of adenosine diphosphate (ADP) to adenosine triphosphate (ATP). Complex I: NADH dehydrogenase; complex II: succinate dehydrogenase; complex III: cytochrome c reductase; complex IV: cytochrome c oxidase; complex V: ATP synthase.

The mitochondrion is the only other source of DNA apart from the nucleus. Mitochondrial DNA (mtDNA) encodes for 22 tRNAs, two rRNAs and 13 polypeptides thought important in the catalytic cores of complexes I, III, IV and V of the oxidative phosphorylation (OXPHOS) system (Fig. 1). In humans, mtDNA contains 16 569 bps and is double stranded.^12^ Depending on the energy needs of a specific tissue, each cell can contain hundreds to thousands of copies of mtDNA.^13^ mtDNA is maternally inherited and has a much higher mutation rate than nDNA, possibly 10 to 17 times higher.^14^ Maternal inheritance results in a lack of bi-parental recombination and therefore the evolution of mtDNA is defined by the emergence of distinct lineages called haplogroups.

Multi-copy makes possible a condition called heteroplasmy, where more than one genotype is present in the same cell/ tissue/organism; homoplasmy then, is where all mtDNA copies carry the same allele. Notably, mtDNA is largely overlooked in GWASs, and could possibly contribute to the missing heredity of CVDs. Next we will consider two main arguments on the possible role of mtDNA variants in CVDs.

## Mitochondrial dysfunction and mtDNA damage in vascular health

When considering mtDNA as a possible contributor to the aetiology of CVD, it should also be considered from a biological perspective. Much investigation has been conducted in an attempt to elucidate the risk factors and physiological mechanisms involved in the development of CVDs, such as sub-clinical atherosclerosis, hypertension, cardiomyopathy and type 2 diabetes.^15^-^20^

**Fig. 2:**
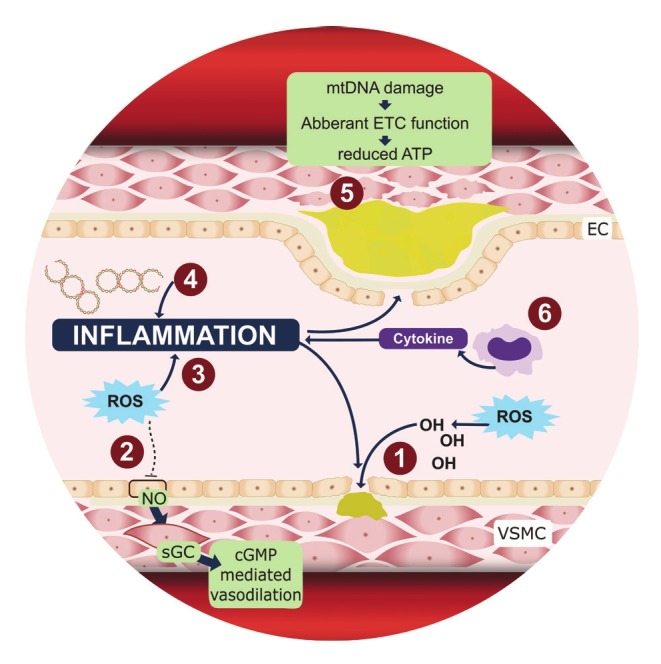
Mitochondrial dysfunction and mtDNA damage affect vascular health in several ways. (1) ROS aids in lesion formation by oxidising lipids, increasing the uptake of inflammatory cells into the vascular wall and enhancing proliferation and hypertrophy in VSMC. (2) During endothelium-dependant vasodilation, EC-released NO activates sGC in VSMC to produce cGMP, signalling a vasodilation response. ROS inhibits this mechanism by quenching bioavailable NO molecules. (3) Endothelial homeostasis is disturbed and plaque formation promoted when mitochondrial dysfunction leads to ROS formation and activates redox-sensitive inflammatory pathways. (4) Circulating cell-free mtDNA is similar in structure to bacterial DNA and invokes an inflammatory response, contributing to atherosclerosis. (5) Independent from ROS formation, mtDNA damage leads to aberrant ETC function and reduced ATP levels in VSMC. When cell viability is compromised, apoptosis of VSMC occurs, accelerating plaque growth and decreasing plaque integrity. (6) Through the same mechanisms, apoptosis of monocytes occurs, releasing inflammatory cytokines, contributing to inflammation and consequently, increasing plaque formation and vulnerability. ATP: adenosine triphosphate; cGMP: cyclic guanosine monophosphate; EC: endothelial cell; ETC: electron transport chain; NO: nitric oxide; ROS: reactive oxygen species; sGC: soluble guanylyl cyclase; VSMC: vascular smooth muscle cells.

An important common feature in all these conditions is inflammation in some form or another (Fig. 2). This inflammatory state is thought to be caused by oxidative stress, due to excessive levels of reactive oxygen species (ROS). ROS can be produced in several pathways, including by enzymes such as NADPH oxidase, nitric oxide synthase, and enzyme complexes of the electron transport chain (ETC).^21^

The general mechanism of ROS involvement in CVDs is ascribed to oxidative effects. For example, ROS contributes to atherosclerotic lesion formation by oxidising lipids, promoting vessel wall uptake of inflammatory cells, and enhancing proliferation and hypertrophy of vascular smooth muscle cells (VSMC).^21^

Several studies have shown increased levels of ROS in hypertensive humans and rats.^16^,^22^,^23^ In cultured VSMCs for example, ROS has been shown to cause changes in cellular signalling pathways, favouring vasoconstriction.^15^ A mechanism for this could be that ROS reduces nitric oxide (NO) bioavailability via quenching, impairing endotheliummediated vasodilation.^21^,^22^,^24^ However, ROS along with other factors of a dysfunctional mitochondrial energy metabolism (e.g. nucleotides, Ca2+) also act as effectors of retrograde signalling and the so-called cell danger response.^25^-^27^

Mitochondria are considered the major producers of ROS within the cell. In a recent article, Lopez-Armada et al.^18^ reviewed the role of mitochondrial dysfunction in the inflammatory response and consequently in the pathology of various diseases, including CVDs. The authors described how mitochondrial dysfunction may modulate inflammatory processes by activating redox-sensitive inflammatory pathways and the NLRP3 inflammasome. In the vasculature, these alterations lead to disturbed endothelial homeostasis, which has been implicated in the pathology of CVDs, such as atherosclerosis.^18^ Indeed, some improvements in disease presentation of hypertension and diabetes have been observed in studies where chronic antioxidant treatment is applied.^18^,^28^,^29^

Another mechanism by which inflammation might be altered by mitochondrial dysfunction is through the resultant release of mtDNA into the cytosol and circulation. Because mtDNA is similar to bacterial DNA and not methylated,^30^ released mtDNA molecules are thought to induce an inflammatory state, which contributes to atherosclerosis and other inflammatory diseases.^31^-^35^

mtDNA damage has also been shown to promote atherosclerosis directly, in the absence of oxidative stress. In a study by Yu et al.,^36^ VSMCs showed increased apoptosis and decreased proliferation in a proof-reading deficient PolG-/-/ ApoE-/- mouse model. Increased secretion of pro-inflammatory factors, tumour necrosis factor-α and interleukin-1β were also reported and implicated in mtDNA release into the cytosol and subsequent activation of the inflammasome.

The authors went on to test the applicability of their findings in humans and concluded that an alternative mechanism for mtDNA defects mediate atherosclerosis development, independent of ROS; mtDNA defects lead to aberrant ECT function and consequently reduce ATP content in VSMCs, which then promote apoptosis and inhibit cell proliferation, leading to increased atherosclerosis and risk of plaque rupture.^36^,^37^ Plaque vulnerability is further promoted by mtDNA defects via monocyte cell death and the resultant increased release of inflammatory cytokine.^38^ From these studies, it can be seen that mitochondrial dysfunction, possibly as a result of mtDNA variants or damage, can directly be implicated in mechanisms that encumber vascular health.

## mtDNA point mutations and cardiac involvement

Clinically proven mtDNA mutations are also an important cause of inherited disease.^39^ To date, more than 250 deleterious point mutations and deletions of the mitochondrial genome have been clinically proven to be associated with certain disease phenotypes (www.mitomap.org). In several of these diseases, cardiovascular symptoms are an important part of the aetiology.

Due to the very high levels of mtDNA population variation seen, both within and between human populations, the identification of mutations causing clinically manifesting disease proves to be a challenge, despite the small size of the mitochondrial chromosome. Initially, DiMauro and Schon had set specific criteria for defining the pathogenicity of mtDNA mutations.^40^ The list has subsequently been updated to include important methods such as functional testing and singlefibre analysis, which can more specifically link genotype to phenotype.^41^,^42^

Notably, a pathogenicity scoring system for mitochondrial tRNAs was devised by McFarland et al.^41^ and further refined by Yarham et al.^43^ Mitchell et al.^44^ also devised a pathogenicity scoring system using variants in complex I mtDNA genes, but this can be applied to any structural mtDNA mutation. A list of these criteria is given in Table 1.

**Table 1:** Criteria for defining the pathogenicity of mtDNA mutations

*Criteria for pathogenicity of mtDNA mutations include*
• The mutation must be present only in patients and not in controls
• The mutation must be present in varied mitochondrial genetic backgrounds
• The mutation must be the best mtDNA candidate variant to be pathogenic
• The mutation must affect functionally important domains
• Transfer of the mutated mtDNA to another cell line must be accompanied by transfer of the cellular or molecular defect
• The mutation must not be a recognised, non-pathogenic, single-nucleotide Polymorphism
• The mutation must alter an area that is known to be highly conserved throughout evolution
• The mutation must occur at varying levels within the cells (i.e. must be heteroplasmic)
• A larger proportion of mutant mtDNA must correspond to a more severe Phenotype
• Single-fibre polymerase chain reaction must be performed by comparing normal and abnormal fibres from muscle
• The secondary structure of the tRNA molecule must also be taken into account when determining mt-tRNA mutation pathogenicity

These criteria need to be met in order for a mtDNA mutation to be classified as ‘disease causing’ for either structural or mt-tRNA mutations.^40^-^43^

It should be noted that there are mtDNA mutations that are exceptions to all the ‘rules’ in Table 1, and this was a critical motivation for algorithms or clinical scoring systems to help weigh the evidence that is presented for each mutation.^43^,^44^ For a clinically proven mutation to manifest as a diseased phenotype, as in the case of primary mitochondrial disorders, the allele frequency (heteroplasmy) needs to exceed a certain threshold, usually above 60%, referred to as the phenotypical threshold effect.^45^

The biochemical threshold effect then refers to the ability of the oxidative phosphorylation (OXPHOS) system to resist the metabolic expression of deficiencies therein.^45^,^46^ These deficiencies may be caused by various factors involved in the expression and regulation of the OXHPOS complexes.

There are many complexities to the expression of mtDNA mutations; a classic example is the mitochondrial tRNA mutation m.3243A>G, the most common of the mtDNA mutations causing mitochondrial disease. The m.3243A>G mutation can result in a vast array of clinical phenotypes affecting multiple systems within the body, causing two distinct clinical syndromes: maternally inherited diabetes and deafness (MIDD), and mitochondrial encephalomyopathy, lactic acidosis, and strokelike episode (MELAS) syndrome in severe cases. Furthermore, the age of onset of m.3243A>G-associated phenotypes spans more than 50 years. The impact of several confounding factors, including heteroplasmy levels, remains unclear.^47^

Another group of well-studied mutations are those that cause the disease Leber’s hereditary optic neuropathy (LHON). In contrast to the m.3243A>G mutation, LHON has a tissuespecific phenotype manifesting as bi-lateral blindness. Several mtDNA mutations have been implicated in LHON, while three of these mutations, namely m.3460G>A, m.11778G>A and m.14484T>C located in subunits ND1, ND4 and ND6 of complex I, respectively, account for 90 to 95% of cases.^48^ Unusually, these mutations can be detected as homoplasmic variants without exerting a phenotype. Rather, disease penetrance is significantly influenced by confounding factors such as gender and environment (clinical penetrance is increased to 93% in smoking men),^49^ and mtDNA haplogroup background (haplogroup J, K and M7 increase risk of clinical penetrance).^50^,^51^

**Fig. 3:**
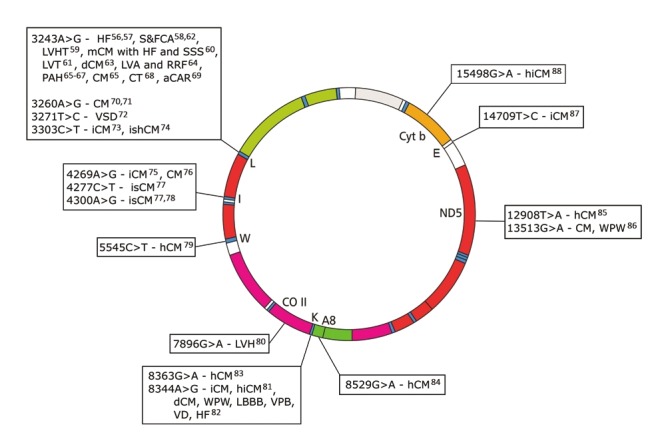
mtDNA morbidity map indicating clinically provenmtDNA mutations that present with syndromic or isolatedcardiac involvement. aCAR: abnormal cardiac autonomicregulation; CM: cardiomyopathy; hCM: hypertrophiccardiomyopathy; dCM: dilated cardiomyopathy;HF: heart failure; hiCM: histiocytoid cardiomyopathy;iCM: infantile cardiomyopathy; ishCM: isolated hypertrophiccardiomyopathy; LBBB: left bundle branch block;LVA: left ventricle abnormalities; LVH: left ventricularhypertrophy; LVHT: left ventricular hyper-trabeculation/non-compaction; mCM: mitochondrial cardiomyopathy;PAH: pulmonary artery hypertension; RRF: raggedred fibres; S&FCA: structural and functional cardiacabnormality; SSS: sick sinus syndrome; VD: ventriculardysfunction; VPB: ventricular premature beats; VSD:ventricular septal defect; WPW: Wolff–Parkinson–Whitesyndrome. See Table 2 for a detailed list of mutations,phenotype, references and pathogenicity scores, asdescribed in Mitchell et al.^44^ and Yarham et al.^43^

The heart has especially high energy needs and relies heavily on OXPHOS-derived ATP, such that one-third of cardiomyocyte volume consists of mitochondria.^52^ Not surprisingly then, the myocardium is frequently affected in primary mitochondrial disorders.^53^ In a retrospective review study by Yaplito-Lee et al.,^54^ 33% of paediatric patients with definitive OXPHOS disorders had cardiac manifestations. Several mtDNA mutations (Fig. 3, Table 2 [online]) have also been shown to exhibit cardiac involvement, either as part of a multi-system syndrome (most frequently seen in MELAS), or as isolated occurrences, such as in the absence of associated CVDs or risk factors thereof.^53^,^55^,^56^

Hypertrophic cardiomyopathy (hCM) and pulmonary artery hypertension (PAH) are the two phenotypes most commonly seen as isolated cardiac manifestations of primary mitochondrial disorders.^53^ If clinically proven mtDNA mutations can directly lead to cardiac dysfunction, is it plausible to think that other mtDNA variants, such as population variants of mildly deleterious effect, might also lead to or alter severity/penetrance of complex cardiovascular disease phenotypes.

From the substantial supportive evidence of mitochondrial involvement in cardiovascular disease, it is therefore evident that genetic investigations on the aetiology of CVD should include consideration of mtDNA variations. In the following sections, we present a number of approaches (plus findings from such investigations) on how mtDNA variation is investigated/ associates in/with disease, with a specific focus on the approaches more likely to show its putative contribution to the risk of CVD development.

## Current approaches used for investigating mtDNA involvement in disease

**Mitochondrial DNA copy number**

mtDNA copy number can be used as an indicative marker of mitochondrial biogenesis, which is thought to increase in response to increased energy demands, such as exercise, but also as a compensatory method for mitochondrial dysfunction.^89^ On the other hand, mtDNA copy number has been shown to decrease with aging,^90^ and has been significantly correlated with late-onset diseases, such as Parkinson’s disease.^91^,^92^ As mentioned above, cell-free circulating mtDNA may also act as an inflammatory agent that contributes to CVDs.^33^

Altered mtDNA copy number measured in peripheral blood cells have been shown to be associated with different complications of diabetes (diabetic retinopathy and diabetic nephropathy).^93^,^94^ Also, an association between telomere length and mtDNA copy number suggests a co-regulatory mechanism for these two parameters, both of which are implicated in aging.^95^ mtDNA depletion and impaired mitochondrial biogenesis have been shown to be a constant factor in the early stages of heart failure^96^,^97^ and other diseases thought to be related to aberrant ROS production.^98^

While the exact mechanisms behind mtDNA content regulation are still unclear, it seems changes in either direction can be causative or indicative of disease.^99^ Measurement of mtDNA copy number can be done accurately by real-time PCR methods, making this a useful approach for investigating the role of mitochondrial metabolism in disease phenotypes.

**Common mtDNA population variants**

mtDNA variants accumulated over time differ between population groups that have been separated for several thousand years. Consequently, distinct lineages (mtDNA haplogroups) can be drawn according to these sets of unique changes in mtDNA, referred to as common population variants. The full human mtDNA phylogeny can be accessed at www.phylotree.org.^100^ Much of the variation seen in modern humans is to be found in the African haplogroups L0 to L6, but this variation has not been as fully described as the variation on other continents. European (e.g. I, J, K, H, T, U, V, W, X) and Asian (e.g. A, B, C, D, F, G) haplogroups fall within super haplogroups M and N, which in turn fall within L3.

mtDNA haplogroup association studies aim therefore to associate these common mtDNA population variants with risk for various complex diseases, such as diabetes, hypertension or Parkinson’s disease.^101^ mtDNA background has been shown to correlate with the severity of cardiomyopathy caused by nDNAencoded mitochondrial protein mutations,^102^ and increases the penetrance of LHON-causing pathogenic mutations.^50^,^51^

It has been proposed that mtDNA population variants could contribute to the adaptability of population groups to their environment by altering mitochondrial enzyme function.^103^,^104^ By analysing non-synonymous variants in 104 complete mtDNA sequences from across the globe, Mishmar et al.^103^ found that the ATP6 and cytochrome b genes were particularly variable in arctic and temperate zones, respectively, leading them to believe that positive selection had taken place. Stressors, such as sudden changes in environment, could then influence the degree of disease susceptibility of these environmentally adapted population groups.^105^ However, this hypothesis was contested by others who have shown that there are significant differences in the same measure in haplogroups from the same environment.^106^,^107^ Additionally, Amo and Brand^108^ put forward evidence to suggest that certain bioenergetic parameters did not significantly differ between mitochondria from arctic versus tropical haplogroups.

In contrast to the action of positive selection, the action of negative or purifying selection on mtDNA has been established for almost a decade.^107^,^109^ One important point to consider is that positive or directional selection could not have acted identically on all lineages, and therefore would result in a different rate of accumulation of variants on haplogroup lineages, thus affecting our ability to time divergence events by counting the mutational events between lineages. On the other hand, it is possible that negative or purifying selection could act evenly across lineages and not impact on our use of mtDNA as a molecular clock; the reliability of mtDNA as a molecular clock has been widely discussed.^110^

Because of the central role that mitochondria play in cell signalling and apoptosis, mitochondria have been implicated in several age-related diseases, including Parkinson’s disease, Alzheimer’s disease, multiple sclerosis and psoriasis.^101^,^111^,^112^ CVDs are also classified as late-onset diseases and mitochondria have also been implicated in CVDs. Consequently, haplogroup association studies on CVD phenotypes are plentiful, but, as will be revealed, also prone to pitfalls.

Crispim et al.^113^ reported an association of European haplogroup cluster J/T with insulin resistance and type 2 diabetes in a Caucasian-Brazilian cohort. On the other hand, Li et al.^114^ found no association between mtDNA variation and risk for developing diabetes, while Chinnery et al.115 found no association with type 2 diabetes and major European haplogroups in a large study using 897 cases and 1 010 controls. Rather, Achilli et al.^116^ found that the risk for developing specific types of diabetes complications (disease outcome) was significantly associated with different mitochondrial haplogroups.

Several mtDNA population variants in cytochrome c oxidase and NADH dehydrogenase subunit genes have been associated with body mass index (BMI) in adults.^117^ In a very large study using a second cohort, Chinnery et al.^118^ found no significant associations between mtDNA haplogroups and ischaemic heart disease, hypertension, diabetes or the metabolic syndrome, but did find a significant association of sub-haplogroup K with risk of cerebral ischaemic vascular effects.

Therefore, while some studies investigating phenotypes included in CVDs have reported results that support a role for mtDNA in CVD,^116^,^117^,^119^,^120^ there are also conflicting reports.^115^,^118^,^121^ This is not only common in CVD-related literature, but all areas where haplogroup association studies have been applied. This is an indication of the many difficulties that need to be overcome when considering mtDNA variation in the context of disease.^122^

The unique way in which mtDNA is inherited (lack of bi-parental recombination), which results in the emergence of numerous unique haplogroups, contributes to the complexity encountered when investigating mtDNA involvement in disease. Non-biological factors such as differences in approach to statistical analysis;^123^ difficulty in proper case and control matching; small effective population size, which results in a higher likelihood of population stratification; and insufficient cohort size,^122^ further undermine the consistency of these studies.

Meta-analysis of data generated by several studies with overlapping phenotypes can be employed to overcome sample size difficulties, but these bring along challenges of their own, as independent studies have different goals/methods, and do not necessarily generate directly comparable datasets.^101^ So, while haplogroup association studies might have fulfilled an important role in the ongoing pursuit of the involvement of mtDNA variation in disease, it is now well recognised that the field needs to consider alternative models.

**Rare mtDNA population variants**

It has been shown that negative or purifying selection plays a significant role in mtDNA evolution, with deleterious variants being removed from the population over time,^107^ and that the power of selection has been equally effective in all human lineages.^124^ Consequently, rare mtDNA population variants are more likely to be mildly deleterious than common variants, as selection has had less time to remove them from the populations. Indeed, rare mtDNA variants have been linked to changes in CVDs and risk factors.

In a study by Govindaraj et al.,^120^ complete mtDNA analysis revealed 10 non-synonymous variants present in hypertrophic cardiomyopathy patients, but not present in controls or on databases. Seven of these variants were classified as likely ‘pathogenic’, using several online scoring tools such as PolyPhen-2, PMUT and PROVEAN, and were therefore thought to be involved in the development of cardiomyopathy. Rare variants m.5913G>A and m.3316G>A have both been suggested to be associated with increased fasting blood glucose levels, while m.5913G>A was shown to also be associated with increased blood pressure in a selected Framingham heart study subset, all of whom were of European descent.^7^

In addition, several rare mtDNA variants, such as m.3316G>A,^7^,^125^ have been implicated in diabetes mellitus, of which an up-to-date list can be found on www.mitomap.org. Another possibility is that the effect of an accumulation of mildly deleterious variants may only become clinically significant once a population is challenged by a rapid change of confounding factors, such as diet or other environmental factors (toxins).^126^,^127^

In conclusion, several approaches are currently in use for investigating the role of mtDNA in common complex disease. mtDNA copy number is an emerging approach that might become more prevalent in studies concerning CVDs as well. In terms of mtDNA variants, rare population variants have been linked to several disease phenotypes, including CVD-related diseases such as cardiomyopathy and diabetes mellitus, and might be found to be associated with other CVDs or risk factors such as hypertension.

Rare population variants are more likely to be mildly deleterious,^124^ but might not have a high enough impact on their own to alter disease onset; rather, these variants might be more likely to alter disease progression or outcomes. For common population variants, several haplogroup association studies have been done in CVDs, but have also been marred by the challenges these types of studies face.^122^ It seems then that an alternative approach to investigating the role of mtDNA variation in disease is needed when investigating common complex disease.

## Alternative approach for investigating mtDNA involvement in disease: the adjusted mutational load hypothesis

An alternative approach, the mutational load hypothesis was put forward in Elson et al.^111^ Mutational load refers to the synergistic effect of several changes in, for example, a specific gene or functionally related set of genes. It does not look for associations with a specific variant but rather a summative effect. While some mtDNA variants might be of little effect on their own, an increased mutational load might be associated with increased risk for a certain disease. mtDNA mutational loads can then be adjusted to reflect the position within the phylogeny, since there are large differences in the average number of common population variants between haplogroups. This approach can also further be modified to, for example, exclude low-impact variants, highlighting the role of likely deleterious functional variants.

Determining the likely impact or pathogenicity of mtDNA variants can be achieved by using several computational pathogenicity-predicting methods.^128^ An example of such a method is the MutPred system, which assigns a MutPred score to any protein-coding mtDNA variant, according to 14 gain/ loss properties of protein structure and function.^129^ The use of this system has been widely validated in the context of mtDNA studies,^124^ and performs better in an accuracy test when compared with several other methods.^128^ Therefore the question can be asked whether individuals in the disease group are impacted on by a combination of rare (mildly deleterious) variants or simply whether such variants are more common in the disease cohort than in the controls.

The mutational load approach moves away from the study of haplogroups and looks at the collective effect of rare (or recent) variants, which are more likely to be deleterious. It distils the likely impact of a person’s mtDNA variation into a single value on a continuous scale rather than a letter. Consequently it will have more statistical power than conventional haplogroupassociation studies, as more powerful parametric statistics can be applied and fewer comparisons are required.^130^ It offers an alternative method to explore the involvement of mtDNA variants in disease phenotypes, including diseases thought to be related to mitochondrial dysfunction, such as CVDs.

## The unique challenges faced by studies in African populations

While communicable diseases are still the leading causes of mortality in sub-Saharan Africa (SSA), CVD particularly is a growing concern here, since the prevalence has risen markedly in recent times as more populations of developing countries become urbanised and are exposed to a diet and lifestyle that increase risk factors for CVD.^131^ Taking into account the many differences among ethnic groups in the onset and development of CVD,^132^,^133^ genomic investigations have also been used to investigate these disparities.^131^,^134^-^136^ However, the number of well-powered genetic studies on CVDs in African populations or people of African descent is much lower than in European populations. As yet, no conclusive nDNA genetic factor/s has been identified to help understand these disparities.^137^

Current Eurocentric reference panels used in GWAS studies to examine the involvement of population variants in disease have been shown to be of limited use in even common SSA population groups.^138^ This is indicative of the lack of African representation in our current databases. This lack extends to mtDNA as well. Of the more than 30 000 mtDNA sequences available on GenBank, only 13% of these are of African lineages (L0–6). This bias in published data results in the resolution of the phylogenetic tree being much higher in the European branches (especially super-haplogroup N descendant) than in the African roots,^139^ despite greater diversity within the latter. Comparatively few studies have been done where the involvement of mtDNA variation in CVD has been considered.^135^,^136^,^140^-^142^

Although of small size, one such study helps to highlight the challenges posed by these gaps in our current data. Ameh et al.^142^ could not find the tRNA mutation m.3243A>G in Nigerian type 2 diabetes patients, despite an association being previously reported in other European and Asian populations. This and other studies^143^ illustrate the difficulty of extrapolating genetic risk factors for disease from one population group to the next, and the need for population-specific studies.

## Conclusion

SSA is facing a growing burden of CVD, while the discrepancies in onset and progression between different ethnicities are still poorly understood. Additionally, there are large data gaps when genetic studies on Africans are considered, especially for complex disease phenotypes. The unique genetic backgrounds of different populations also make it difficult to apply advances made in well-studied populations to understudied populations.

While great efforts are being made to address these data gaps by initiatives such as the Human Heredity and Health in Africa (H3Africa) initiative,^131^ the Southern African Human Genome Programme, and the African Genome Variation Project,^138^ there is an urgent need for even more large-scale African-specific investigations (which should also consider mtDNA variation) to be undertaken if we are to provide the necessary care to all vulnerable groups.^144^

Realistically, for some time still, it is likely that studies in African populations will be hampered by financial and logistic/infrastructural difficulties,^144^ limiting the sizes thereof. Fortunately, these studies can benefit from retrospective lessons we have learned thus far in other populations, highlighted in the above discussions. New studies could particularly benefit by asking better-formulated questions, and using alternative approaches that aim to address the challenges associated with many of the classic approaches used, when the role of mtDNA in common disease is investigated.

**Key messages**

- Cardiovascular disease (CVD) is a leading global cause of morbidity and mortality, and its incidence is on the rise in sub-Saharan Africa.
- Discrepancies in the onset and progression of CVDs exist between different ethnic and population groups, which nuclear genetic studies have so far failed to explain. Mitochondrial DNA (mtDNA) offers a viable alternative target for genetic studies concerning common complex disease.
- Many approaches can be taken to investigate the role of mtDNA in disease, but not all are suited for studies influenced by moderate cohort size or population stratification. The adjusted mutational load hypothesis offers an alternative approach, which could be of particular value for much-needed studies on CVDs in under-represented sub-Saharan African populations.
